# The Correlation between Elevated HDL-Cholesterol, Body Mass Index, and Presence of Thyroid Nodules: A Retrospective Analysis

**DOI:** 10.3390/jcm12237411

**Published:** 2023-11-29

**Authors:** Cafer Zorkun, Kenan Yalta, Alara Eren, Ertan Yetkin

**Affiliations:** 1 Department of Cardiology, Faculty of Medicine, Istanbul University, 34093 Istanbul, Turkey; 2 Department of Cardiology, School of Medicine, Trakya University, 22030 Edirne, Turkey; 3 Istanbul Faculty of Medicine, Istanbul University, 34093 Istanbul, Turkey; 4 Cardiology Clinic, Turkiye Hospital, 34381 Istanbul, Turkey

**Keywords:** atherosclerosis, cardiovascular protection, HDL-cholesterol, body mass index, thyroid nodules

## Abstract

Background: Elevated high-density lipoprotein-cholesterol (HDL-cholesterol) levels have been linked to unfavorable outcomes in various clinical settings, but the association with thyroid nodules remains unclear. We aimed to analyze the correlation between elevated HDL-cholesterol and the presence of thyroid nodules along with certain demographic and clinical findings. Methods: In this retrospective study, the patients were divided into three groups based on their body mass index (BMI): <25, 25–29, and >30 and evaluated. Data of 677 patients aged between 15 and 95 years (52.6 ± 15.6) were evaluated. The entire study population comprised 516 females (76.2%). Results: Thyroid nodules (67.1%) and left ventricle diastolic dysfunction (LVDD) (58.1%) were the two most frequent findings in the overall cohort. In the multivariate regression model, BMI, heart rate, and HDL-cholesterol values were significant and independent predictors (*p* = 0.000 for all) of the presence of thyroid nodules. The presence of thyroid nodules is higher in females, particularly within the higher BMI groups [odds ratio (OR) = 1.048 (CI = 1.02–1.08) for BMI < 25, *p* = 0.003; OR = 1.094 (CI = 1.05–1.14) for BMI 25–29, *p* = 0.000; OR = 1.115 (CI = 1.05–1.19) for BMI ≥ 30]. This higher incidence is not observed in males. Conclusion: While the precise mechanisms underlying this association are yet to be fully elucidated, elevated HDL-cholesterol may serve as an indicator of thyroid nodules rather than a marker of cardiovascular protection.

## 1. Introduction

Current information based on numerous interventions, epidemiological research, and Mendelian randomization studies has suggested that higher levels of high-density lipoprotein-cholesterol (HDL-cholesterol) are associated with a lower cardiovascular risk. This concept is based on the generally favorable effects of HDL-cholesterol on the cardiovascular system. HDL-cholesterol particles, known for their role in the reverse cholesterol transport pathway, also play a crucial role in the innate immune system [1,2,3]. They contribute to cardiovascular health by facilitating reverse cholesterol transport and exhibiting anti-inflammatory, antioxidant, antithrombotic, endothelial repair, angiogenesis, and antidiabetic effects [1,2,3,4,5,6,7,8]. On the other hand, little is clearly known about the pathophysiological and clinical implications of abnormally elevated HDL-cholesterol levels in the clinical setting. Elevated levels of HDL-cholesterol are oddly linked to an increased risk of death from all causes, including cardiovascular and cancer-related deaths, in both men and women [9]. What is significant about this association is that it forms a U-shaped and non-linear pattern, indicating that having both low and extremely high levels of HDL-cholesterol could be harmful due to various mechanisms [9,10,11,12]. However, the mechanisms by which elevated HDL-cholesterol levels act in a detrimental fashion are still vague. One such mechanism could be the emergence of a dysfunctional HDL-cholesterol phenotype at elevated HDL-cholesterol concentrations [9], leading to the negation of the favorable effects of HDL-cholesterol with a shift to augmented inflammation and associated oxidative pathways [10]. More specifically, HDL-cholesterol serves as a transporter of certain pro-inflammatory mediators, including serum amyloid A [10]. Research examining the functional and structural characteristics of HDL-cholesterol in cardiovascular and renal diseases has been progressively challenging the long-held belief that HDL-cholesterol is a good cholesterol [11,12].

Over recent decades, thyroid nodules, which are the most common thyroid disorders and develop due to an interplay of genetic, environmental, or endogenous factors, have been increasingly reported in the general population [13]. Although most patients with thyroid nodules do not exhibit clinical symptoms, these nodules are often linked to various disorders, including endocrine dysregulation, autoimmune thyroid disease, altered body composition, and a range of metabolic abnormalities. They also have poorly understood pathogenetic implications [13,14]. Moreover, the association of thyroid nodules with serum thyroid hormones and thyroglobulin levels significantly varies [13], making these markers unreliable predictors of existing thyroid nodules. Therefore, widely available and stable biochemical markers are required to help determine the need for thyroid imaging in the presence of vague signs and symptoms of thyroid nodules. In this context, HDL-cholesterol could serve as an epitome of such markers due to its widespread availability and stable characteristics, such as the absence of abrupt fluctuations in serum. However, the association between excessive HDL-cholesterol levels and thyroid nodules remains to be established. To the best of our knowledge, there exists no report exclusively focusing on this association in the literature. Accordingly, we aimed to analyze the impact of elevated HDL-cholesterol levels on the incidence of thyroid nodules along with certain demographic and clinical findings.

## 2. Materials and Methods

### 2.1. Study Population

At the cardiology outpatient clinic, we encountered an intriguing pattern among patients who had complained of weakness and fatigue—some of them exhibited elevated HDL-cholesterol levels. As we delved deeper into their medical histories, we found that many of these patients led sedentary lifestyles, characterized by a lack of exercise and being overweight. Surprisingly, the numbers of such cases were significant. This observation prompted us to take the matter to the ethics committee, sparking our decision to conduct further research on this intriguing phenomenon.

The inclusion criteria for this study were as follows: not having a diagnosed rheumatic or autoimmune disease, whether it was in an active or remissive state; having a diagnosed thyroid nodule; being overweight; not using any known medication that affected HDL-cholesterol metabolism; not having hypothyroidism or autoimmune thyroiditis; not engaging in regular exercise beyond their daily routine, in other words, having a sedentary lifestyle; and/or having elevated HDL-cholesterol levels. Exclusion criteria for this study included the use of thyroid medications, corticosteroids, insulin, phenytoin, estrogen, and statins, as well as a history of thyroid dysfunction or thyroid surgery, alcohol intake, cirrhosis, known gene mutations potentially associated with changes in HDL-cholesterol metabolism, and a history of excessive exercise. Finally, data of 677 patients (516 female, 76.2%) aged between 15 and 95 years (mean age, 52.6 ± 15.6 years) who were examined at our clinics between October 2010 and June 2012 were retrospectively evaluated. 

The study was conducted in accordance with the Declaration of Helsinki and approved by the Institutional Review Board of Istanbul Yedikule Chest Diseases and Surgery Education and Training Hospital (07.09.2012/4336) and with no request for informed consent for the evaluation, processing, or presentation of anonymized patient-related information.

### 2.2. Blood Tests, Measurements, and Radiological Imaging

Records of imaging data (e.g., transthoracic echocardiography, thyroid ultrasonography, etc.) and laboratory tests for lipid profiles served as essentials in the evaluation. Data related to patient demographics, medical background, physical examination findings (heart rate (HR), blood pressure (BP), body mass index (BMI)), and other laboratory parameters were also extracted from the hospital’s archiving system. BMI was calculated by dividing the weight in kilograms by the square of the height in meters. The formula used for calculating BMI was BMI = weight (kg)/[height (m)]^2^. The patients were divided into three groups based on their body mass index: <25, 25–29, and >30 and evaluated.

### 2.3. Statistical Analysis

The descriptive statistics included mean, standard deviation, median, minimum, maximum values, frequency, and percentage. The distribution of variables was assessed using the Kolmogorov–Smirnov test. The Mann–Whitney U test was employed for comparing quantitative data, while the Chi-square test was utilized for analyzing qualitative data comparisons. Logistic regression and Receiver Operating Characteristic (ROC) analyses were performed to demonstrate the effect level, and a Forest plot was employed for a subgroup analysis. A *p*-value of <0.05 at the 95% CI was regarded as statistically significant. Statistical analyses were conducted using SPSS 28.0.

## 3. Results

The entire study population comprised 516 females (76.2%) and 161 males (23.8%). The demographic, clinical, and laboratory data are presented in Table 1. Thyroid nodules were diagnosed in 454 patients (67.1%), coronary artery disease (CAD) in 70 patients (10.4%), diabetes mellitus (DM) in 246 patients (36.3%), hypertension (HT) in 289 patients (42.7%), left ventricular diastolic dysfunction (LVDD) in 393 patients (58.1%), family history of CAD (FHCAD) in 306 patients (45.2%), and cigarette smoking in 327 patients (48.3%). Thyroid nodules (67.1%) and LVDD (58.1%) were the two most frequent findings in the overall cohort.

Patients were categorized into two groups based on the presence of thyroid nodules (Table 2). The incidence of thyroid nodules appeared to be higher in females, patients with HT, and patients with LVDD (*p* values: 0.000, 0.001, and 0.000, respectively). However, the incidences of CAD, DM, family history of coronary artery disease, and cigarette smoking were comparable between the groups. 

The serum concentrations of total cholesterol, HDL-cholesterol, and low-density lipoprotein-cholesterol (LDL-cholesterol) significantly differed between the groups (*p* = 0.000 for all). The Mann–Whitney U test exhibited significant differences in age, BMI, and HR between the groups (*p* = 0.000 for all; Table 2). 

In the univariate model, significant effects of age, sex, BMI, LVDD, heart rate, total cholesterol, HDL-cholesterol (*p* = 0.000 for all), hypertension (*p* = 0.001), folic acid (*p* = 0.013), LDL (*p* = 0.020), triglycerides (*p* = 0.041), and fT3 (*p* = 0.029) values were observed in patients with and without thyroid nodules. In the multivariate regression model, BMI, heart rate, and HDL-cholesterol values were significant and independent predictors (*p* = 0.000 for all) of the presence of thyroid nodules (Table 3).

When differentiating between patients with or without thyroid nodules, the significant effectiveness of HDL-cholesterol levels was observed (AUC = 0.797 (0.763–0.832)) (Table 4).

In female patients, the significant effectiveness of HDL-cholesterol values (AUC = 0.871 (0.839–0.903)) was observed in distinguishing patients with or without thyroid nodules. However, in male patients, the discriminatory effect of HDL-cholesterol values was not significant for those with or without thyroid nodules (AUC = 0.550 (0.461–0.639)) (Table 4).

In the entire group with a BMI < 25, no significant discriminatory effect of HDL-cholesterol values was observed in distinguishing patients with or without thyroid nodules. In the BMI 25–29 group, the significant effectiveness of HDL-cholesterol values (AUC = 0.797 (0.734–0.859)) was observed in distinguishing patients with or without thyroid nodules. In the BMI ≥30 group, the significant effectiveness of HDL-cholesterol values (AUC = 0.878 (0.812–0.944)) was observed in distinguishing patients with or without thyroid nodules (Table 4).

In females with a BMI < 25, no significant discriminatory effect of HDL-cholesterol values was observed in distinguishing patients with or without thyroid nodules (AUC = 0.546 (0.411–0.681), *p* = 0.000). In females with a BMI 25–29, the significant effectiveness of HDL-cholesterol values (AUC = 0.810 (0.723–0.896), *p* = 0.000) was observed in distinguishing patients with or without thyroid nodules. In females with a BMI ≥ 30, the significant effectiveness of HDL-cholesterol values (AUC = 0.881 (0.804–0.958), *p* = 0.000) was observed in distinguishing patients with or without thyroid nodules (Table 4).

In males with a BMI < 25 (AUC = 0.463 (0.319–0.607), *p* = 0.615), BMI 25–29 (AUC = 0.558 (0.422–0.694), *p* = 0.404), and BMI ≥ 30 (AUC = 0.685 (0.469–0.900), *p* = 0.113), no significant discriminatory effect of HDL-cholesterol values was observed in distinguishing patients with or without thyroid nodules (Table 4).

**Figure 1:**
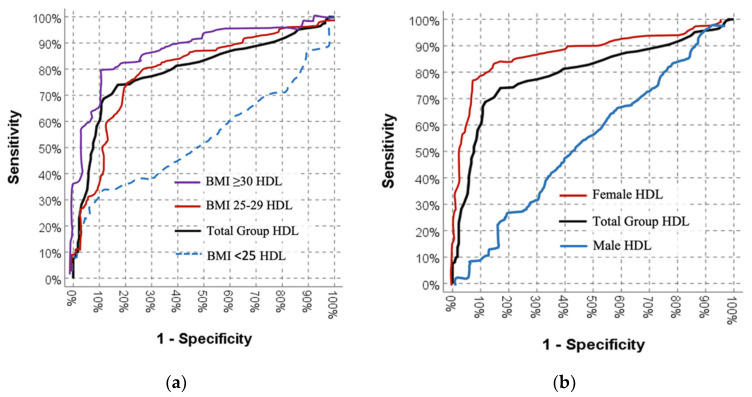
Receiver Operating Characteristic (ROC) curves: (**a**) for HDL/HDL-cholesterol vs. BMI (**left**) and (**b**) sex vs. HDL/HDL-cholesterol (**right**). (BMI: body mass index, HDL/HDL-cholesterol: high-density lipoprotein-cholesterol.)

Figure 1 illustrates the relationship between HDL-cholesterol levels and the presence of thyroid nodules in the entire group, with a focus on the association with BMI (left), as well as separate analyses for males and females (right). Out of the 161 males, thyroid nodules were detected in 79 patients (49.07%) with elevated HDL-cholesterol levels, but higher HDL-cholesterol levels did not predict the presence of thyroid nodules in males. In contrast, among the 516 females, 375 (72.67%) had thyroid nodules. Elevated HDL-cholesterol levels strongly indicated the presence of thyroid nodules in females.

**Figure 2:**
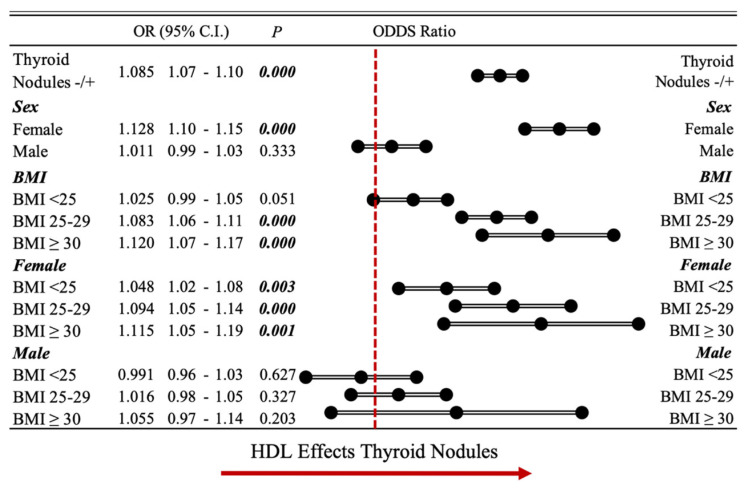
Interpretation of study results with Forest plot graphic. Presence of thyroid nodules was higher in females, particularly within the higher BMI groups (BMI: body mass index, HDL/HDL-cholesterol: high-density lipoprotein-cholesterol).

Additionally, the Forest plot graphic in Figure 2 demonstrates that the presence of thyroid nodules was higher in females, particularly within the higher BMI groups [OR 1.048 (CI: 1.02–1.08) for a BMI < 25, *p* = 0.003; 1.094 (CI: 1.05–1.14) for a BMI 25–29, *p* = 0.000; 1.115 (CI: 1.05–1.19) for a BMI ≥ 30]. This higher incidence was not observed in males.

## 4. Discussion

According to the current medical literature and common belief, individuals with high HDL-cholesterol levels are thought to have a lower susceptibility to cardiovascular diseases. While reports have indicated that elevated HDL-cholesterol levels may accompany undesirable clinical conditions, the relationship with the presence of thyroid nodules has not been addressed. In the current analysis, the most notable finding appears to be the independent association between elevated HDL-cholesterol levels, compared with the group average, and an increased incidence of thyroid nodules. From a mechanistic perspective, excessive HDL-cholesterol levels may induce tumor growth by triggering systemic inflammation. 

Importantly, augmented systemic inflammation has a pivotal impact on the synthesis and release of certain growth factors, including galectin, transforming growth factor β and a variety of angiogenic factors [15]. Therefore, systemic inflammation and associated growth factors may also have an important pathogenetic role in thyroid nodules. Certain infections, including Helicobacter pylori and chronic viral hepatitis, elicit a significant propensity for the evolution of the thyroid nodules, possibly through the upregulation of specific mediators, including CXCL10 and tumor necrosis factor-α in the thyroid gland [16,17,18]. More interestingly, systemic inflammation may impair the synthesis of thyroid hormones with consequent increases in thyroid-stimulating hormones (TSHs) associated with tumoral growth in thyroid tissue [16]. Of note, TSH levels were not found to be significantly elevated in those with an existing thyroid nodule in the current analysis. However, even though the mean TSH levels measured at a time remained within the reference values or insignificantly elevated, increased temporal TSH compared with the baseline may have also been of pathogenetic significance and should also be taken into consideration in this setting. On the other hand, available data did not allow for the sequential evaluation of TSH levels in the current analysis. Therefore, large-scale prospective studies are strongly warranted to investigate the implications of TSH in the evolution of thyroid nodules associated with excessive HDL-cholesterol levels. Taken together, higher HDL-cholesterol levels may have been associated with the presence and/or evolution of thyroid nodules, possibly through a detrimental impact on systemic inflammation and associated factors. This notion was recently corroborated in a population of 2722 subjects with metabolic syndrome who exhibited a higher prevalence of thyroid nodules, which were mostly attributed to increased TSH levels and systemic inflammation [19]. However, higher HDL-cholesterol levels and associated factors may manifest in females, suggesting sex-specific characteristics, which was exclusively demonstrated in females in the current analysis. Therefore, the female hormonal milieu appeared to be a prerequisite for the abovementioned mechanisms associated with systemic inflammation, at least, in the setting of thyroid pathologies. This also held true for increased BMI, which exclusively served as a risk factor in the female population. These notions may have also been substantiated by the independent impact of the female sex on thyroid nodule evolution (approximately 2-fold). However, the independent impact of the hormonal milieu associated with the female sex seemed much weaker in this setting compared with those of HDL-cholesterol levels and associated factors. Interestingly, current information is extremely scarce as to how and to what extent hyperlipidemia is associated with thyroid nodule evolution. One study reported hyperlipidemia as a significant risk factor in a population of 309,576 patients during a 10-year follow-up [20]. 

Upon a comprehensive assessment, this study achieved a notably homogeneous group by omitting individuals who were on thyroid medications, corticosteroids, insulin, phenytoin, estrogen, and statins. This exclusion also extended to those with a background of thyroid disorders or surgery, alcohol consumption, cirrhosis, or any known genetic mutations that may have affected HDL-cholesterol metabolism, as well as those with a pattern of excessive exercise. The primary reason for the lower mean HDL-cholesterol values recorded in this study, compared with those in the existing literature, could be attributed to these specific exclusions.

In our analysis, BMI, higher HDL-cholesterol levels, and the incidence of LVDD were significantly higher in females, possibly due to sex-specific hormones. Therefore, we were able to suggest LVDD as an independent risk factor (about 2.88-fold), hypertension (about 1.76-fold), and BMI (about 1.33-fold). LDL-cholesterol had no significant impact on this. The reason for the weaker impact of LDL-cholesterol compared with that of HDL-cholesterol in this setting remains to be fully established; however, it may have been due to the relatively modest implication of certain factors, such as systemic inflammation. We hold the opinion that this impact may have served as an epiphenomenon (as a bystander condition, a consequence of enhanced systemic inflammation or altered thyroid hormonal status, etc.) rather than as a direct trigger of thyroid neoplasia. Interestingly, the protective impact of smoking may have arisen as a coincidental phenomenon in the current analysis and could not be advocated by scientific means. Finally, these findings were in line with the current literature suggesting that heart failure with preserved ejection fraction was more frequent in females and had certain risk factors, including obesity and DM [21]. These findings carry a variety of clinical implications. 

The primary implication could involve screening overweight and/or female patients who have higher HDL-cholesterol levels for possible thyroid nodules. Additionally, when elevated HDL-cholesterol levels are present, family screening may be warranted to identify genetic predispositions. These findings could also hold significant cardiovascular implications, necessitating diagnostic and therapeutic considerations. Due to the inclusion and exclusion criteria, as well as the differences in study group characteristics and methodology, the average HDL-cholesterol values in this study may not have been parallel to those previously published in the literature [22,23,24]. In previous studies, the inclusion criteria for patients were different. For example, individuals with excessive alcohol consumption (72–240 g per week for men; 24–132 g per week for women) were examined in the case of extremely high HDL-cholesterol [23,24]. In our study, however, alcohol consumption was one of the exclusion criteria (because it increases HDL-cholesterol) [22]. Individuals with known autoimmune thyroiditis were also not included in this study. Similarly, the patients evaluated in this study were those who did not have diagnosed rheumatic or autoimmune diseases (whether in active disease or in remission), did not use medications known to affect HDL-cholesterol metabolism, did not have hypothyroidism, and did not engage in regular exercise beyond their daily routine mobility. These exclusion criteria could be one reason why the higher HDL-cholesterol values detected in the patients included in this study may not have been as high as those referenced in the literature.

Interestingly, in chronic diseases or during infections, HDL-cholesterols may not only lose their protective roles but could also adopt harmful functions [25]. It has been observed that the relationship between HDL-cholesterol levels and various conditions is U-shaped, indicating that both very low and excessively high levels of HDL-cholesterol are linked to a heightened risk of numerous diseases and higher mortality rates [25,26,27,28]. 

Due to the ineffectiveness of intervention trials, HDL-cholesterol, unlike LDL-cholesterol, has not been established as a target for treatment. Nonetheless, HDL-cholesterol remains a key factor in assessing the risk of ASCVD, both directly and indirectly, as it is used in calculating non-HDL-cholesterol and even LDL-cholesterol [11,25]. Particularly in asymptomatic patients not undergoing any lipid-modifying therapy, a low HDL-cholesterol level is regarded as a risk factor for ASCVD development. Consequently, HDL-cholesterol is included in most clinical risk prediction models endorsed by guidelines for ASCVD prevention. However, these models and current algorithms for assessing cardiovascular disease risk often fail to acknowledge the non-linear relationship between HDL-cholesterol levels and risk. This oversight leads to a lowered estimation of risk in individuals with very high HDL-cholesterol levels, an area in need of improvement [11,12,25]. Additionally, the previously held belief regarding the ‘protective role of high HDL-cholesterol levels’ needs reassessment; high HDL-cholesterol should no longer be seen as a protective factor, a stance of significant importance [25,29]. It is time for clinical laboratories, clinicians, and practitioners to move away from the widespread practice of calculating total cholesterol/HDL-cholesterol or LDL-cholesterol/HDL-cholesterol ratios, which risks underestimating the threat posed by high HDL-cholesterol [29]. Contrary to earlier assumptions, high HDL-cholesterol levels do not necessarily imply a lower risk of atherosclerotic cardiovascular disease. In fact, they may indicate a similar or increased risk compared with moderate levels and are linked to higher risks of infections and premature mortality. Thus, high HDL-cholesterol levels should not be reassuring when other risk factors, such as LDL hypercholesterolemia, are present [29,30].

The dialogue regarding the therapeutic implications of elevated HDL-cholesterol levels is in its early stages [29]. The causal relationship between high HDL-cholesterol and increased mortality, as well as risks for chronic kidney disease, infectious diseases, Alzheimer’s disease, or age-related macular degeneration, remains uncertain. As previously noted, alcohol consumption could be a significant contributing factor [22]. Additionally, results from a previous epidemiological study indicated that [24], sex differences across countries, and in various populations, also played a significant contributing role in HDL-cholesterol levels. Major observational studies have suggested that very high HDL-cholesterol levels are linked to cardiovascular and other mortalities. This may have been due to several factors: rare genetic variants leading to dysfunctional HDL-cholesterol that affects cholesterol removal, high alcohol consumption potentially causing increased mortality, inefficient cholesterol transport to the liver (such as slowed catabolism) leading to oversized HDL-cholesterol particles that could accumulate in arteries and contribute to atherosclerosis and atherosclerotic cardiovascular diseases, and poor health possibly resulting in abnormally high HDL-cholesterol levels [25]. Considering all of this, it may be necessary to redefine the term “excessive or abnormally high HDL-cholesterol”. Given the lack of effective treatments for reducing HDL-cholesterol, it is recommended to focus on managing other established risk factors in patients with high HDL-cholesterol, similar to the approach for patients with low HDL-cholesterol [22,24,26,29,30].

The current study had a variety of inherent limitations that should be noted. First, the retrospective study design could be deemed a limitation; however, there existed no clinical rationale to prospectively perform thyroid ultrasonography in every patient. Second, the impact of confounding factors (e.g., iodine deficiency, radiation, etc.) on the evolution of thyroid nodules could not be fully eliminated. Accordingly, further similar studies, particularly in geographical areas where these factors are less prevalent, seem necessary to deem HDL-cholesterol an absolute risk factor in this context. Third, the study did not provide absolute mechanistic insights regarding HDL-cholesterol levels in the study population. Moreover, the study focused on the quantity (and not the quality) of HDL-cholesterol. Therefore, the impact of dysfunctional HDL-cholesterol on the evolution of thyroid nodules remains speculative. Fourth, the study did not provide specific information on the nature of existing thyroid nodules (e.g., benign, or malignant subtypes). Therefore, the differential impact of HDL-cholesterol on various thyroid pathologies could not be thoroughly categorized. In particular, the relationship between thyroid cancers (detected upon further examination of nodules) and high HDL-cholesterol levels may have been even stronger due to the well-known role of systemic inflammation in cancer. Fifth, the number of included male and female subjects was not equal. As this study followed a retrospective design, we analyzed data from all available female and male patients who met our specified criteria. It is important to note that the number of male patients meeting these criteria was comparatively lower. Nevertheless, we comprehensively assessed the data of all male patients accessible within the same time frame. Sixth, our database included complete blood counts (hemograms). However, the timing of these tests was not uniformly distributed, meaning they were not conducted at the same time. As a result, we did not include the evaluation of neutrophil–lymphocyte or platelet–lymphocyte ratios in our analysis. Instead, we focused on assessing cholesterol profiles and thyroid ultrasound results, as these were performed close to each other in time, ensuring a reliable and simultaneous comparison without any doubts. Finally, even though systemic inflammation might have important pathogenetic implications in this setting, specific inflammation markers could not be evaluated due to the retrospective design of the study. On the other hand, C-reactive proteins (CRPs) were available only in a portion of patient data and were not taken into consideration due to their non-specific nature. 

## 5. Conclusions

This study’s results suggested that higher HDL-cholesterol levels should not be seen as protection against coronary artery disease, especially in overweighted and obese women. Instead, it pointed to a possible link between higher HDL-cholesterol levels and the presence of thyroid nodules in this group. However, this connection was not found in males. Investigating the precise mechanisms responsible for this correlation necessitates further exploration. It is essential to note that these findings were preliminary, emphasizing the need for validation through additional clinical studies.

## Figures and Tables

**Table 1:** General characteristics of study group represented as numbers and proportion (CAD: coronary artery disease, DM: diabetes mellitus, Fe: iron, FeBC: iron binding capacity, fT3: free T3, fT4: free T4, HT: hypertension, LVDD: left ventricular diastolic dysfunction, FHCAD: family history of coronary artery disease, Fe: iron, FeBC: iron binding capacity, TSH: thyroid stimulating hormone, VLDL-C: very low-density lipoproteins-cholesterol).

		Min-Max			Median	Mean ± Sd/*n*-%		
Age (y)		15.0	-	95.0	53.0	52.6	±	15.6
Sex	Female					516		76.2%
	Male					161		23.8%
Height (Cm)		142.0	-	188.0	165.0	164.6	±	5.6
Weight (Kg)		50.0	-	114.0	76.0	74.3	±	10.1
BMI (Kg/m^2^)		17.2	-	46.8	28.0	27.4	±	3.6
BMI (Kg/m^2^)	<25					203		30.0%
	25–29					326		48.2%
	≥30					148		21.9%
Cigarette Smoking	(−)					350		51.7%
	(+)					327		48.3%
CAD	(−)					607		89.7%
	(+)					70		10.3%
DM	(−)					431		63.7%
	(+)					246		36.3%
Hypertension	(−)					388		57.3%
	(+)					289		42.7%
LVDD	(−)					284		41.9%
	(+)					393		58.1%
FHCAD	(−)					371		54.8%
	(+)					306		45.2%
Thyroid Nodules	(−)					223		32.9%
	(+)					454		67.1%
Heart Rate (bpm)		51.0	-	97.0	66.0	67.7	±	7.4
Glucose (mg/dL)		35.0	-	443.0	98.0	106.2	±	35.7
Uric Acid (mg/dL)		1.7	-	13.7	4.7	5.0	±	1.6
Total Cholesterol (mg/dL)		111.0	-	504.0	205.0	210.6	±	50.4
Triglycerides (mg/dL)		26.0	-	2657	118.5	148.3	±	141.8
HDL-cholesterol (mg/dL)		24.0	-	144.0	58.0	59.3	±	16.6
LDL-cholesterol (mg/dL)		13.0	-	313.0	122.0	126.0	±	41.7
VLDL (mg/dL)		5.0	-	531.0	23.0	29.8	±	33.9
FE (mcg/dL)		7.0	-	231.0	71.0	73.8	±	35.7
FeBC (mcg/dL)		51.0	-	458.0	277.0	283.4	±	68.8
Ferritin (mcg/dL)		2.4	-	216.0	23.5	39.7	±	41.2
Folic Acid (ng/mL)		1.8	-	32.0	7.1	8.0	±	4.1
B12 (mg/dL)		71.0	-	1500	239.0	288.3	±	182.1
fT3 (pg/mL)		1.9	-	12.5	2.9	3.0	±	0.6
fT4 (ng/mL)		0.2	-	2.9	0.9	0.9	±	0.2
TSH (μIU/mL)		0.0	-	100.0	1.5	2.3	±	5.0

**Table 2:** Comparison of patients with and without existing thyroid nodules (Mann–Whitney U and Chi-square test results; CAD: coronary artery disease, DM: diabetes mellitus, Fe: iron, FeBC: iron binding capacity, fT3: free T3, fT4: free T4, HT: hypertension, LVDD: left ventricular diastolic dysfunction, FHCAD: family history of coronary artery disease, Fe: iron, FeBC: iron binding capacity, TSH: thyroid stimulating hormone, VLDL-cholesterol: very low-density lipoprotein-cholesterol).

		Thyroid Nodules (−)				Thyroid Nodules (+)				*p*	
		Mean ± Sd/*n*-%			Median	Mean ± Sd/*n*-%			Median		
Age (y)		48.6	±	14.7	48.0	54.6	±	15.6	55.0	0.000	^m^
Sex	Female	141		63.2%		375		82.6%		0.000	^X2^
	Male	82		36.8%		79		17.4%			
Height (cm)		165.6	±	6.0	166.0	164.1	±	5.3	165.0	0.004	^m^
Weight (Kg)		69.6	±	11.5	67.0	76.6	±	8.4	78.0	0.000	^m^
BMI (Kg/m^2^)		25.3	±	3.8	24.1	28.5	±	3.0	28.7	0.000	^m^
BMI (Kg/m^2^)	<25	132		59.2%		71		15.6%		0.000	^X2^
	25–29	65		29.1%		261		57.5%			
	≥30	26		11.7%		122		26.9%			
Cigarette Smoking		119		53.4%		208		45.8%		0.065	^X2^
CAD		21		9.4%		49		10.8%		0.581	^X2^
DM		80		35.9%		166		36.6%		0.861	^X2^
Hypertension		75		33.6%		214		47.1%		0.001	^X2^
LVDD		91		40.8%		302		66.5%		0.000	^X2^
FHCAD		105		47.1%		201		44.3%		0.490	^X2^
Heart Rate (bpm)		72.2	±	7.5	71.0	65.5	±	6.3	64.0	0.000	^m^
Glucose (mg/dL)		105.8	±	26.5	100.0	106.4	±	39.5	97.0	0.021	^m^
Uric Acid (mg/dL)		5.0	±	1.6	4.8	4.9	±	1.6	4.7	0.514	^m^
Total Cholesterol (mg/dL)		200.3	±	46.9	193.0	215.6	±	51.3	215.0	0.000	^m^
Triglycerides (mg/dL)		166.2	±	126.2	128.0	139.6	±	148.2	115.0	0.000	^m^
HDL-cholesterol (mg/dL)		48.4	±	10.9	47.0	64.7	±	16.3	65.0	0.000	^m^
LDL-cholesterol (mg/dL)		120.5	±	38.7	115.0	128.7	±	42.9	128.0	0.008	^m^
VLDL-cholesterol (mg/dL)		31.4	±	21.2	25.0	28.9	±	38.8	22.0	0.005	^m^
FE (mcg/dL)		74.3	±	37.3	71.0	73.6	±	34.8	72.0	0.943	^m^
FeBC (mcg/dL)		283.5	±	73.1	277.0	283.4	±	66.4	275.7	0.974	^m^
Ferritin (mcg/dL)		46.8	±	47.7	29.6	35.6	±	36.5	22.7	0.078	^m^
Folic Acid (ng/mL)		7.2	±	3.2	6.5	8.5	±	4.5	7.4	0.018	^m^
B12 (mg/dL)		280.3	±	197.5	225.5	292.9	±	173.0	246.0	0.158	^m^
fT3 (pg/mL)		3.05	±	0.58	3.00	2.91	±	0.63	2.90	0.000	^m^
fT4 (ng/mL)		0.88	±	0.20	0.85	0.88	±	0.21	0.86	0.569	^m^
TSH (μIU/mL)		2.7	±	7.3	1.7	2.2	±	3.4	1.5	0.088	^m^

^m^ Mann–Whitney U test/^X²^ Chi-square test.

**Table 3:** Univariate and multivariate logistic regression analysis demonstrating female sex, BMI, left ventricular diastolic dysfunction, abnormally elevated high-density lipoprotein levels as independent predictors of existing thyroid nodules (LVDD: left ventricular diastolic dysfunction, BMI: body mass index, fT3: free T3, HDL-cholesterol: high-density lipoprotein-cholesterol, LDL-cholesterol: low-density lipoprotein-cholesterol, OR: odds ratio, CI: confidence interval).

	Univariate Model			Multivariate Model		
	OR	95% CI	*p*	OR	95% CI	*p*
Age (y)	1.025	1.015–1.037	0.000			
Sex	0.362	0.252–0.522	0.000	0.462	0.218–0.981	0.044
BMI (Kg/m^2^)	1.334	1.260–1.412	0.000	1.189	1.119–1.262	0.000
Hypertension	1.760	1.261–2.456	0.001			
LVDD	2.882	2.071–4.011	0.000			
Heart Rate (bpm)	0.872	0.848–0.896	0.000	0.950	0.904–0.999	0.045
Total Cholesterol (mg/dL)	1.007	1.003–1.010	0.000			
Triglycerides (mg/dL)	0.999	0.997–1.000	0.041			
HDL-cholesterol (mg/dL)	1.085	1.069–1.101	0.000	1.065	1.033–1.099	0.000
LDL-cholesterol (mg/dL)	1.005	1.001–1.009	0.020			
Folic Acid (ng/mL)	1.096	1.020–1.177	0.013			
fT3 (pg/mL)	0.659	0.453–0.957	0.029			

Logistic Regression (Forward LR).

**Table 4:** The table for the AUC analysis shows the relation between HDL-cholesterol, sex, and BMI (AUC: area under the curve, BMI: body mass index, CI: confidence interval, HDL-C/HDL-cholesterol: high-density lipoprotein-cholesterol).

		AUC	% 95 Confidence Interval	*p*
Total Group HDL-C		0.797	0.763–0.832	0.000
Gender	Female Group HDL-C	0.871	0.839–0.903	0.000
	Male Group HDL-C	0.550	0.461–0.639	0.278
BMI (Kg/m^2^)	<25 Group HDL-C	0.527	0.436–0.619	0.523
	25–29 Group HDL-C	0.797	0.734–0.859	0.000
	≥30 Group HDL-C	0.878	0.812–0.944	0.000
Female BMI (Kg/m^2^)	<25 Group HDL-C	0.546	0.411–0.681	0.422
	25–29 Group HDL-C	0.810	0.723–0.896	0.000
	≥30 Group HDL-C	0.881	0.804–0.958	0.000
Male BMI (Kg/m^2^)	<25 Group HDL-C	0.463	0.319–0.607	0.615
	25–29 Group HDL-C	0.558	0.422–0.694	0.404
	≥30 Group HDL-C	0.685	0.469–0.900	0.113

## Data Availability

The data presented in this study are available on request from the corresponding author. The data are not publicly available due to [institutional restrictions].
